# The impact of a minor child’s cancer diagnosis on parental work: a scoping review

**DOI:** 10.1590/1980-220X-REEUSP-2026-0175en

**Published:** 2026-10-02

**Authors:** Shuyuan Shi, Yuqi Jiang, Hongyan Zhao, Wen Lu, Yuwen Zhou, Yujie Guo

**Affiliations:** 1 Nantong University, School of Nursing and Rehabilitation, Nantong, China.; 2 Taizhou Second People’s Hospital Affiliated to Yangzhou University, Taizhou, China.

**Keywords:** Child, Neoplasms, Work, Parents, Scoping Review, Criança, Neoplasias, Trabalho, Pais, Revisão de Escopo

## Abstract

**Objective:**

This study aims to systematically analyze the impact of having a minor child with cancer on parents’ work, including the influencing factors and dynamic adjustment strategies.

**Method:**

The study used Arksey and O’ Malley framework and PRISMA-ScR list to guide the review process and minimize potential bias. The Ecological Model of Health was adopted as the theoretical framework to analyze the influencing factors extracted from the included studies. We searched PubMed, Excerpt Medica Database (Embase), Web of Science, Cumulative Index to Nursing and Allied Health Literature (CINAHL), and the Cochrane Library databases for articles published from the date of database inception to December 31, 2025. Two researchers performed article screening and data extraction.

**Results:**

A total of 8,236 articles were retrieved, of which 21 met the inclusion criteria. Guided by the Ecological Model of Health, this study demonstrates that parental work is influenced by the interplay of multiple factors. Parental work adjustments are dynamic and may include sick leave, changes in work hours, resignation, or job changes.

**Conclusion:**

When a child is diagnosed with cancer, parents’ work is often significantly affected. Personalized workplace interventions and stronger policy support are therefore needed to help them balance work and caregiving responsibilities.

## RESUMO

**Objetivo:**

Este estudo tem como objetivo analisar sistematicamente o impacto, no trabalho dos pais, do diagnóstico de câncer em um filho, incluindo os fatores de influência e as estratégias de adaptação dinâmica.

**Método:**

O estudo utilizou a estrutura de Arksey e O’Malley e a lista PRISMA-ScR para orientar o processo de revisão e minimizar o potencial viés. O Modelo Ecológico de Saúde foi adotado como referencial teórico para analisar os fatores de influência extraídos dos estudos incluídos. Realizamos buscas nas bases de dados PubMed, Excerpt Medica Database (Embase), Web of Science, Cumulative Index to Nursing and Allied Health Literature (CINAHL) e Cochrane Library por artigos publicados desde a data de criação das bases de dados até 31 de dezembro de 2025. Dois pesquisadores realizaram a triagem dos artigos e a extração de dados.

**Resultados:**

Foram recuperados 8.236 artigos, dos quais 21 atenderam aos critérios de inclusão. Guiado pelo Modelo Ecológico de Saúde, este estudo demonstra que o trabalho parental é influenciado pela interação de múltiplos fatores. Os ajustes no trabalho parental são dinâmicos e podem incluir licença médica, alterações no horário de trabalho, pedido de demissão ou mudança de emprego.

**Conclusão:**

Quando uma criança recebe o diagnóstico de câncer, o trabalho dos pais costuma ser afetado de maneira significativa. São, portanto, necessárias intervenções personalizadas no local de trabalho e um apoio de políticas mais robusto para ajudá-los a conciliar o trabalho e as responsabilidades de cuidados.

## INTRODUCTION

With the development of early cancer screening and treatment technologies, both the incidence and survival rates of minor children with cancer have increased^(1)^. According to the World Health Organization (WHO), approximately 400,000 children are diagnosed with cancer each year^(2)^, and the global incidence rate continues to rise^(3)^. After being diagnosed with cancer, minors typically undergo a long treatment cycle, accompanied by significant side effects^(4)^. During this process, pediatric cancer patients are often highly dependent on the continuous care, emotional support, and financial security provided by their parents, which can have a significant, multidimensional, and lasting impact on their parents’ social lives^(5,6)^. For example, financially, the family’ s financial burden increases significantly, with 63% of families facing the dual pressures of soaring treatment costs and declining income^(7)^. Regarding mental health, parents’ physical and emotional well-being is also significantly affected, with anxiety and depression levels notably increasing during treatment^(8)^. In terms of sleep, caregiving minor children with cancer often consumes a large amount of parents’ time and energy, with 72% of caregivers reporting severe sleep deprivation^(9)^.

These multifaceted influences intertwine and collectively affect parents’ work. The diagnosis of cancer in a child can lead to work interruptions for parents, reduced working hours, hindered work development, and even the risk of exiting the labor market altogether^(10)^. For instance, parents need to take sick leave or reduce their working hours to provide ongoing medical care for their child. Previous studies have shown that parental work stability plays a crucial role in the treatment and recovery of children with cancer^(11,12)^. Once parents’ work is affected, the continuity and predictability of household income decline, exacerbating the financial strain from medical expenses and thereby limiting access to and sustained investment in treatment related resources^(13)^. At the same time, role conflict and financial strain from work instability can increase parents’ psychological burden, weaken family function, and disrupt caregiving continuity. This may lead to adverse effects on family members’ mental health, children’s physical rehabilitation, and their long-term quality of life^(14)^.

However, no research has yet systematically revealed which factors influence the work of parents whose minor children have cancer, nor what work adjustments parents make at different stages of their children’s treatment. Existing literature has primarily focused on non-work-related impacts, mainly discussing parental psychological stress^(15)^, caregiving burdens^(16)^, and financial toxicity^(17)^, with factors directly related to work appearing in a fragmented manner. Furthermore, there is a lack of cohesive dialogue and integration across studies regarding specific work adjustments made by parents, leading to scattered and inconclusive findings. As a result, no comprehensive evidence has been developed to guide practice and policy. To address this gap, we conducted a scoping review aimed at systematically summarizing the existing evidence on how childhood cancer affects parents’ work, identifying the factors influencing their work, and exploring the work adjustments parents adopt after their child’s cancer diagnosis.

## METHOD

Given the broad nature of the research topic, this scoping review was conducted systematically in accordance with established methodological guidance for scoping reviews^(18)^ and reported following the Preferred Reporting Items for Systematic Reviews and Meta-Analyses extension for Scoping Reviews (PRISMA-ScR)^(19)^. Additionally, Arksey and O’Malley’s five stage methodological framework was employed to guide the review process and minimize potential bias^(20)^. It should be noted that due to the characteristics of scoping reviews, methodological quality evaluation is not required and, consequently, was not performed for the included studies. In addition, the protocol was registered at the Centre for Open Science (https://doi.org/10.17605/OSF.IO/BHSJA).

### Identification of Research Questions

(1) What factors influence parents’ work after their minor child is diagnosed with cancer?
(2) What work adjustments do parents make after their minor child is diagnosed with cancer?

### Search Strategy

We systematically searched PubMed, Excerpt Medica Database (Embase), Web of Science, Cumulative Index to Nursing and Allied Health Literature (CINAHL), and the Cochrane Library databases for articles published from the date of database inception to December 31, 2025. The search strategy incorporated both subject headings and keywords. An example of the detailed search strategy implemented in PubMed is provided in Table 1. Reference lists of included studies were examined, and citation tracking was performed to identify additional relevant studies.

**Table 1:** Search strategy for PubMed –– Multíples países, 2000–2025.

No.	Search Strategy	Records, n
1	((((((“Child”[Mesh]) OR (Minor*[Title/Abstract])) OR (Adolescent*[Title/Abstract])) OR (infant*[Title/Abstract])) OR (teenager*[Title/Abstract])) OR (children[Title/Abstract])) OR (teen*[Title/Abstract])	3,238,829
2	((((“Neoplasms”[Mesh]) OR (cancer*[Title/Abstract])) OR (tumor*[Title/Abstract])) OR (neoplasm[Title/Abstract])) OR (malignan*[Title/Abstract])	5,381,295
3	((((((“Family”[Mesh]) OR “Parents”[Mesh]) OR “Caregivers”[Mesh]) OR (fami*[Title/Abstract])) OR (parent*[Title/Abstract])) OR (carer*[Title/Abstract])) OR (father[Title/Abstract])) OR (mother[Title/Abstract]) OR (family caregivers[Title/Abstract]) OR (informal caregivers[Title/Abstract])	2,159,156
4	(((((((((“Work”[Mesh]) OR (Job[Title/Abstract])) OR (Employment[Title/Abstract])) OR (Occupations[Title/Abstract])) OR (Unemployment[Title/Abstract])) OR (Income[Title/Abstract])) OR (Work Engagement[Title/Abstract])) OR (workplace[Title/Abstract])) OR (work market[Title/Abstract])) OR (Work-Life Balance[Title/Abstract])	429,894
5	#1 AND #2 AND #3 AND #4	1,181

Abbreviations: MeSH, Medical Subject Headings. The filters used were as follows: “full text,””English,””2000/01/01–2025/12/31.”

### Eligibility Criteria

The research question was defined using the PCC (Population, Concept, Context) principle^(21)^: (1) Population: parents of minor children diagnosed with cancer; (2) Concept: the impact of minor children with cancer on parental work, including influencing factors and dynamic adjustment strategies; (3) Context: Studies from various countries and healthcare settings, considering different socioeconomic statuses, work environments, and cultural contexts.

### Inclusion and Exclusion Criteria

Inclusion criteria: (1) Parents of children and adolescents diagnosed with cancer ≤18 years; (2) Research content explicitly involving the impact of the work situation of parents of minor children with cancer; (3) Study design including original research employing qualitative, quantitative, or mixed methods (including longitudinal or cross-sectional studies); (4) Language of the publication being English; (5) Published in peer-reviewed journals between 2000 and 2025.

Exclusion criteria: (1) Conference abstracts; (2) Studies that solely described the psychological and health impacts on parents of children with cancer without addressing work-related consequences; (3) Studies that the full text could not be obtained through database links, institutional access, journal websites, reference checking, and online full-text searches.

### Study Selection and Data Extraction

All retrieved literature records were uploaded to Endnote X9 for literature management and identification of duplicates, which were removed before screening. Records that needed to be screened were imported into Rayyan (https://www.rayyan.ai/) for blinded title and abstract screening. Two reviewers independently screened all titles and abstracts against the predefined eligibility criteria. If there were any disagreements, they consulted and discussed with a third researcher until a consensus was reached. Additionally, the reference lists of included studies were manually screened to identify further eligible publications. Data extraction was independently conducted by two reviewers using a pre-designed data extraction form. The data collected included the first author, year of publication, country, study design, sample size, parental work adjustments, the child’s clinical status, and factors influencing parental work.

To systematically organize and synthesize the factors affecting parental work, the Ecological Model of Health was adopted as the analytical framework, and the identified factors were classified across multiple levels. This model, proposed in health promotion research, posits that health-related outcomes and behavioral changes are not solely determined by individual characteristics, but rather by the combined effects of multiple factors related to the individual and their social environment^(22)^. This model is adopted primarily for two reasons. First, it is widely used in health and cancer-related research, guiding multi-level interventions, particularly in cancer care, and emphasizing systemic support from the individual to the policy level^(23,24)^. Second, the model provides a clear hierarchical classification and judgment logic, helping to incorporate previously scattered work-related factors into a unified classification system, thereby forming a comprehensive and comparable structured picture of evidence. All data extraction and classification results were discussed by the research team and finalized through consensus.

## RESULTS

### General Information of Included Literature

A total of 8,236 articles were identified in the initial search. After removing 1,601 duplicates, the titles and abstracts of 6,635 articles were screened, resulting in the exclusion of 6,553 articles. The remaining 82 articles were assessed according to the eligibility criteria. Ultimately, 21 eligible articles were included in the review. The detailed study selection process is shown in Figure 1.

**Figure 1:**
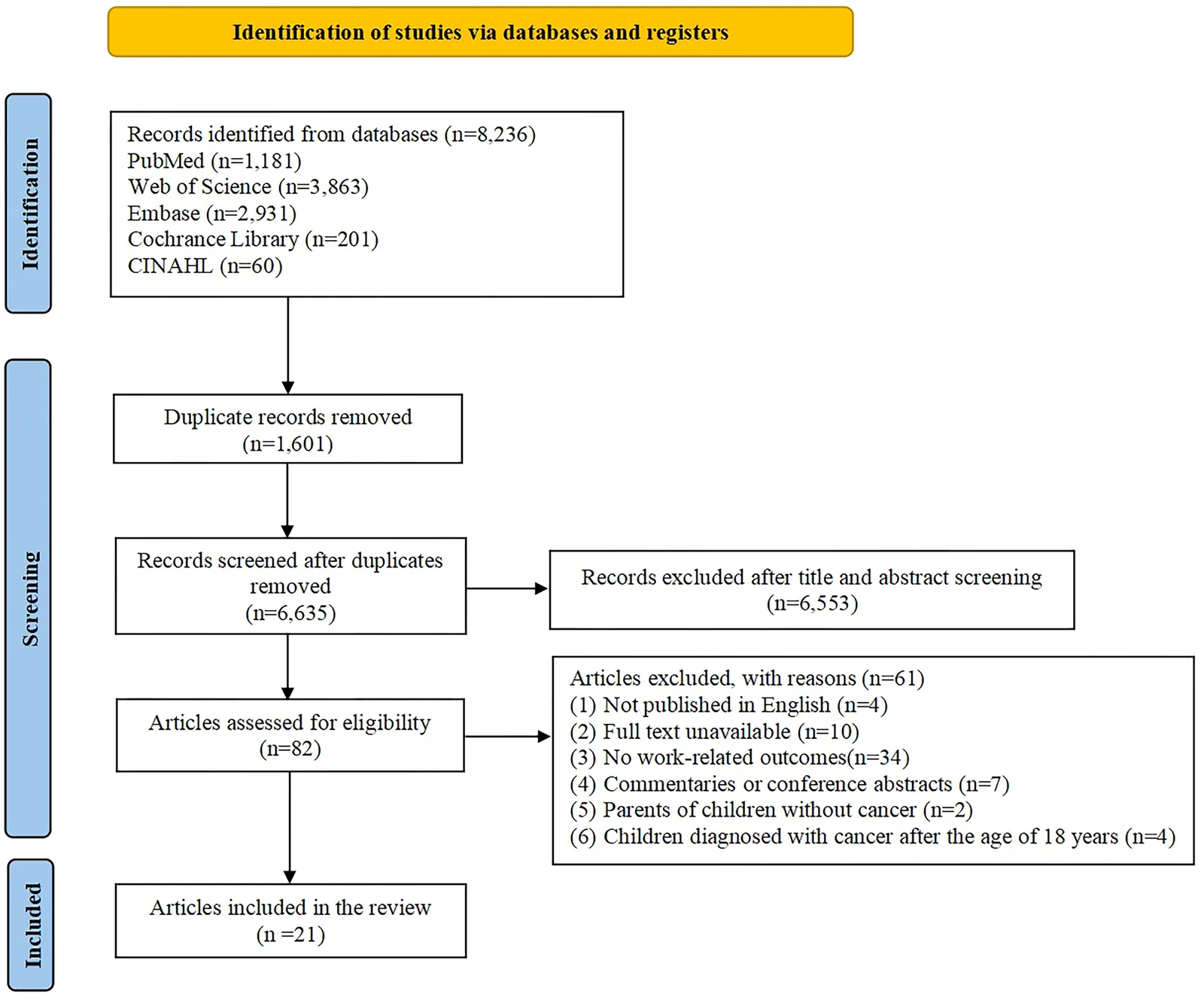
Flowchart of literature screening.

The included studies were conducted in ten countries: the United States, Australia, Germany, Sweden, Switzerland, Denmark, Japan, South Korea, Canada, and Finland. Among the 21 studies, five of them were retrospective cohort studies, three were prospective cohort studies, five were cross-sectional studies, seven were qualitative studies, and one was a retrospective case analysis. These studies were published between 2004 and 2025 and focused on parents of children aged 0 to 18 years who had been diagnosed with cancer. The timeframes examined spanned from the point of diagnosis through the treatment process, the end of the treatment phase, and the period following the child’s death, to explore the impact of these different stages on parental work.

The total sample size of this scoping review was 14,009 parents of children with cancer. Among the included studies, the one by Goodenough et al.^(25)^ reported only the number of families, not individual parents, and was therefore excluded from the total sample size calculation. Additionally, the studies by Lindahl Norberg^(26)^ and Hjelmstedt^(27)^ both utilized data from the Swedish Childhood Cancer Registry (SCCR), involving the same group of participants (n = 3,626). To avoid double counting, this sample was included only once in the overall total. The basic information of the included literature is shown in Table 2.

**Table 2:** General information of the included studies – Multíples países, 2000–2025.

Authors and country	Article types	Characteristics of parents of children with cancer	Characteristics of Children with Cancer	Description of results
Ospelt et al.^(10)^ Switzerland	Cross-sectional study	276 mothers (M ± SD = 37.3 ± 5.9 years, range 21 – 57 years) 193 fathers (M ± SD = 39.9 ± 6.5 years, range 23 – 64 years)	Age at diagnosis: ≤18 years. At least 5 years had passed since diagnosis.	267 mothers: 70 reported changes in their employment, including 33 who reduced their working hours, 23 who resigned, 13 who took unpaid leave, 3 who changed jobs, and 1 who increased working hours. 193 fathers: 28 reported changes in their employment, including 17 who reduced their working hours, 3 who resigned, 7 who took unpaid leave, 2 who changed jobs, 2 who increased their working hours, and 1 who became unemployed.
Hammer et al.^(28)^ Denmark	Retrospective cohort study	1,609 parents whose child died from cancer (mothers: n = 857, Median [IQR] = 37.5 [33.2 – 42.4] years, range 19.8–60.3 years), (fathers: n = 752, Median [IQR] = 39.7 [34.8 – 45.6] years, range 22.9–63.8 years).	Age at diagnosis: ≤18 years. Died before the age of 30 years (Median = 8. 3 years). One year before the child’s death or index date until 5 years after.	Cancer-bereaved Mothers: Overall increased risk of long-term sick leave (HR = 1.62; 95% CI:1.48–1.77), with the strongest effect observed in the first 2 years following the child’s death (HR = 3.74; 95% CI: 3.25–4.31). Lower odds of working in the first 2 years following the loss (OR = 0.44; 95% CI: 0.39–0.49), with a mean of 15 months of work in the first 2 years compared to 18 months for unexposed mothers. Increased risk of unemployment (HR = 1.53; 95% CI: 1.37–1.70), with the strongest effect in the first 2 years post-loss (HR = 1.95; 95% CI:1.69–2.26). Cancer-bereaved Fathers: No overall increased risk for sick leave, but an increased risk for long-term sick leave in the first 2 years post-loss (HR = 2. 10; 95% CI:1.67–2.63), and a slightly lower risk after 5 years (HR = 0.81; 95% CI: 0.67–0.96). Lower odds of working (OR = 0.65; 95% CI: 0.53–0.79), with a mean of 21 months of work compared to 22 months for unexposed fathers. Increased risk for permanently reduced work ability (HR = 1.29; 95% CI: 1.01–1.66).
Okada et al.^(29)^ Japan	Cross-sectional study	171 mothers (Median [IQR] = 44 [41 – 49] years)	Age at diagnosis: (Median [IQR] = 4 [1–8] years). Age at diagnosis:≤15 years. Time since the completion of cancer treatment for their children: (M [IQR] = 60 [24–114] months).	At the time of diagnosis, 59.6% of mothers were still employed. Among these, 40.0% of regular employees and 18.6% of non-regular employees took extended leave, while 36.0% of regular employees and 58. 1% of non-regular employees quit their jobs. At the time of the survey, 75.4% of mothers were employed. Among them, 48.5% were non-regular employees, 21.6% were regular employees, and 5.3% were self-employed.
Yi et al.^(30)^ Korea	Qualitative study	20 fathers (M ± SD = 41.35 ± 4.49 years, range 32–49 years)	Age at diagnosis: (M ± SD = 6.35 ± 4.08 years, range = 1 – 16 years). Within 5 years of the diagnosis.	In the early stages of treatment: Mothers need to take care of their children around the clock, while fathers need to balance other jobs. Fathers will leave work early to spend more time with their children.
Hjelmstedt et al.^(31)^ Sweden	Qualitative study	21 mothers and 11 fathers (Median = 44 years, range 31–51 years)	Age at diagnosis: ≤18 years. Years since diagnosis, (Median = 5 years, range = 1 – 16 years).	Parents would rather focus all their energy on their children, but to manage the family finances, they had to continue working or return to work as soon as possible. Some parents said that because they were too focused on taking care of their children, they were fired by their employers, their wages were delayed, and even their careers were stagnant.
Peikert et al.^(32)^ Germany	Qualitative study	29 mothers (M ± SD = 42 ± 7. 9 years, range = 26–65 years) 20 fathers (M ± SD = 43. 4 ± 5. 9 years, range = 31–52 years)	Age at diagnosis: (M ± SD = 5.5 ± 4.5 years, range = 0 – 17 years). About 16 – 24 months after intensive cancer treatment.	In more than half of the families, the child’s illness caused at least one parent to take sick leave during treatment. 35% of parents reduced their working hours, stopped working, or chose a career with better working conditions. Two parents lost their jobs as a result.
Hjelmstedt et al.^(33)^ Sweden	Retrospective cohort study	1, 394 mothers (M ± SD = 36.75 ± 6.89 years) 1, 394 fathers (M ± SD = 39.36 ± 7.44 years)	Age at diagnosis: ≤18 years. One year before diagnosis to seven years after diagnosis.	When a child is diagnosed with cancer, parents’ TPL (temporary parental leave) increases dramatically. Within one year of their child’s diagnosis, mothers and fathers of children with cancer had an average TPL of 48.3 days and 32.2 days, respectively, compared with an average TPL of 3.3 days and 2.6 days for mothers and fathers without cancer, respectively. Five years after diagnosis, mothers with cancer still had significantly higher rates of TPL than parents without cancer, while fathers with cancer still had significantly higher rates of TPL six years after diagnosis. Among parents of children with cancer, mothers had more TPL days than fathers from one year before diagnosis to five years after diagnosis.
Kelada et al.^(34)^ Australia	Qualitative study	50 mothers and 6 fathers (M ± SD = 40.21 ± 6.3 years, range = 20 – 55 years)	Age at diagnosis: (M ± SD = 5.17 ± 4.72 years, range = 0 – 18 years). Years since diagnosis (M ± SD = 2.89 ± 2.26 years). Years since treatment completion (M ± SD = 2.13 ± 2.04 years).	Twenty-nine parents (51.8%) reported that they and/or their partners reduced their working hours or quit their job after their child was diagnosed. Mothers appeared more likely than fathers to stop working completely. Participants and their partners reduced their working hours by taking unpaid leave (13, 23.2%), taking extended paid leave (including sick leave and long service leave) (5, 8.9%), switching from full-time to part-time or casual work (6, 10.7%), changing working hours for self-employment (3, 5.4%), quitting their job (12, 21.4%), or a combination of these.
Tsimicalis et al.^(35)^ Canada	Qualitative study	78 parents of children with cancer (mothers: M ± SD = 37.83 ± 7.95 years), (fathers: M ± SD = 40.31 ± 7.60 years)	Age at diagnosis: (M ± SD = 7.72 ± 5.4 years, range = 0 – 18 years). Diagnosed with cancer within the previous 21 days.	One or both parents increase their paid working hours to compensate for income.
Hjelmstedt et al.^(27)^ Sweden	Retrospective cohort study	1, 881 mothers (M ± SD = 36.8 ± 7 years) 1, 745 fathers (M ± SD = 39.5 ± 7.6 years)	Age at diagnosis: ≤18 years. One year before diagnosis to seven years after diagnosis.	The risk of sick leave was statistically significantly higher up to six years following a child’s cancer diagnosis. The increase in number of days with sickness benefit was most pronounced the year after diagnosis. Bereavement was associated with a heightened risk of sick leave, especially on the year of the child’s death.
Lindahl Norberg et al.^(26)^ Sweden	Retrospective cohort study	1, 881 mothers (M ± SD = 36.8 ± 7 years) 1, 745 fathers (M ± SD = 39.5 ± 7.6 years)	Age at diagnosis: ≤18 years. One year before diagnosis to seven years after diagnosis.	Compared to mothers in the control group, mothers were less likely to continue working after their children were diagnosed with cancer. This effect was significant both in the year of diagnosis and for up to five years afterward.
Wikman et al.^(36)^ Sweden	Prospective cohort study	152 parents of children with cancer, including 77 mothers and 75 fathers. (M ± SD = 38.88 ± 6.35 years) 42 bereaved parents of children with cancer, including 22 mothers and 20 fathers (M ± SD = 37.19 ± 7.48 years).	Age at diagnosis: ≤18 years. Children with cancer who have been treated for cancer in the past 5 years.	At T7, there were no differences in the proportion working or taking sick leave among surviving mothers and fathers (92% vs. 96% working, 20% vs. 18% taking sick leave), nor among bereaved mothers and fathers (91% vs. 90% working, 14% vs. 20% taking sick leave). The proportion of fathers working did not change from T6 to T7 (91% vs. 96% of surviving fathers, 95% vs. 90% of bereaved fathers). The proportion of working was higher among surviving mothers (92% vs. 82%) and bereaved mothers (91% vs. 77%) at T7 compared with T6, this increase was not significant. The proportion of bereaved mothers taking sick leave was lower at T7 than at T6 (14% vs. 59%, p < 0. 05). More fathers reported taking sick leave at T7 than at T6 (18% vs. 8% for survivors and 20% vs. 15% for bereaved fathers), this was not significant.
Mader et al.^(37)^ Switzerland	Cross-sectional study	379 mothers (M ± SD = 42.7 ± 4.8 years) 378 fathers (M ± SD = 45.8 ± 5.8 years)	Age at diagnosis: (M ± SD = 3. 1 ± 2. 5 years). Time since diagnosis: (M ± SD = 9. 0 ± 2. 5 years).	Mothers of children with cancer have a higher rate of unemployment, while fathers have a higher rate of full-time employment.
Lindahl Norberg et al.^(26)^ Sweden	Retrospective cohort study	1, 881 mothers (M ± SD = 36.8 ± 7 years) 1, 745 fathers (M ± SD = 39.5 ± 7. 6 years)	Age at diagnosis: ≤18 years. One year before diagnosis to seven years after diagnosis.	Mothers were less likely to continue to work after their children were diagnosed with cancer than control group mothers. This effect was evident in the year of diagnosis and up to 5 years after diagnosis. Parents’ incomes declined significantly after their children’s cancer diagnosis. This effect was most pronounced in mothers, whose incomes continued to decline for six years after diagnosis, while fathers’ incomes reached parity with those in the control group within three years of diagnosis.
Goodenough et al.^(25)^ Australia	Retrospective case-note analysis	104 families	Age at diagnosis: (Mean = 7 years). 2 weeks to 2 years after cancer diagnosis. Time since diagnosis: (Mean = 584 days).	1. In the sample of 104 families, 58% showed negative changes in parental paid employment, with an average loss of 53% in family income. 2. About 80% of working mothers stopped working or reduced their hours, while only one-third of working fathers stopped working or reduced their hours, with the peak of change occurring within six weeks of a cancer diagnosis.
Okada et al.^(38)^ Japan	Cross-sectional study	60 mothers	Age at diagnosis: (M ± SD = 4. 9 ± 4. 2 years). Time since diagnosis: (M ± SD = 4. 4 ± 2. 8 years).	Of the 32 mothers who worked at the time of diagnosis, 10 continued to work, 12 took an extended leave, and 10 quit working, and 70% lost motivation for work following diagnosis.
Wakefield et al.^(39)^ Australia	Qualitative study	44 mothers (M ± SD = 42.5 ± 6.7 years, range 29 – 64 years) 34 fathers (M ± SD = 45.9 ± 8.5 years, range 31 – 65 years)	Age at diagnosis: (M ± SD = 13.3 ± 2.6 years, range 7 – 17 years). Within 5 years of the diagnosis and completion of treatment.	Compared to before their children were diagnosed with cancer, parents are often willing to reduce their working hours and accept lower salaries in exchange for more time with their families.
Hovén et al.^(40)^ Sweden	Prospective cohort study	139 mothers (M ± SD = 37.2 ± 6.6 years) 138 fathers (M ± SD = 39.8 ± 6.8 years)	Age at study: (M ± SD = 8.2 ± 5.2 years, range 0-18 years). From one week after diagnosis to one year after completion of treatment.	The highest rates of parental unemployment and sick leave occurred during the period their children were receiving treatment. Compared to at the time of diagnosis, the number of mothers still working three months after treatment decreased, while the number of mothers taking sick leave increased one year after treatment.
Fletcher et al.^(41)^ Canada	Qualitative study	9 mothers (range 32 – 47 years)	Age at diagnosis: range 6 months to 17 years. Children under 17 years of age who have been treated for cancer in the past 5 years; Only one child was receiving treatment at the time of the study, and four had died.	Three women had to adjust their work schedules; three had to take unpaid leave; one woman was on maternity leave when she was diagnosed but was fired for not returning to work after her maternity leave ended; and one woman had to resign. One woman’s husband quit his job to take care of the children.
Bennett Murphy et al.^(42)^ America	Cross-sectional study	20 mothers and 20 fathers	Age at diagnosis: range 18 months to 18 years. Children with cancer were at least 4 months post-diagnosis.	Of the 20 mothers, 8 continued to work. All fathers of children with cancer worked an average of 48 hours per week and spent 4.5 hours per day caring for their children. (Fathers in the control group averaged 42.5 hours per week and spent 4 hours per day with their children)
Lähteenmäki et al.^(43)^ Finland	Prospective cohort study	24 mothers (range = 25 – 49 years) 19 fathers (range = 32 – 48 years)	Age at diagnosis: (Mean = 4.5 years, range 0.1 – 15 years). The first year after their child’s cancer diagnosis.	In the first few months after their child’s cancer diagnosis, the proportion of mothers with children who worked outside the home decreased. Three months after the diagnosis, 33% of mothers said they had stopped working simply because of their child’s illness.

Note:

T6(one year after end of successful treatment or 18 months after transplantation/child’s death)

T7(five years after end of successful treatment or a child’s death)

TPL (temporary parental leave)

### Factors Influencing Parental Work After their child’s Cancer Diagnosis

Currently, no research has summarized and categorized the influencing factors of a minor’s cancer diagnosis on their parents’ work. To address this issue, this study introduces the Ecological Model of Health as an analytical framework. Based on this framework, this review classifies the impact of a minor child’s cancer diagnosis on the parents’ work into five levels: personal traits, psychological and behavioral characteristics, interpersonal relationships, living and working conditions, and policy environment. Relevant factors identified in the literature are shown in Figure 2.

**Figure 2:**
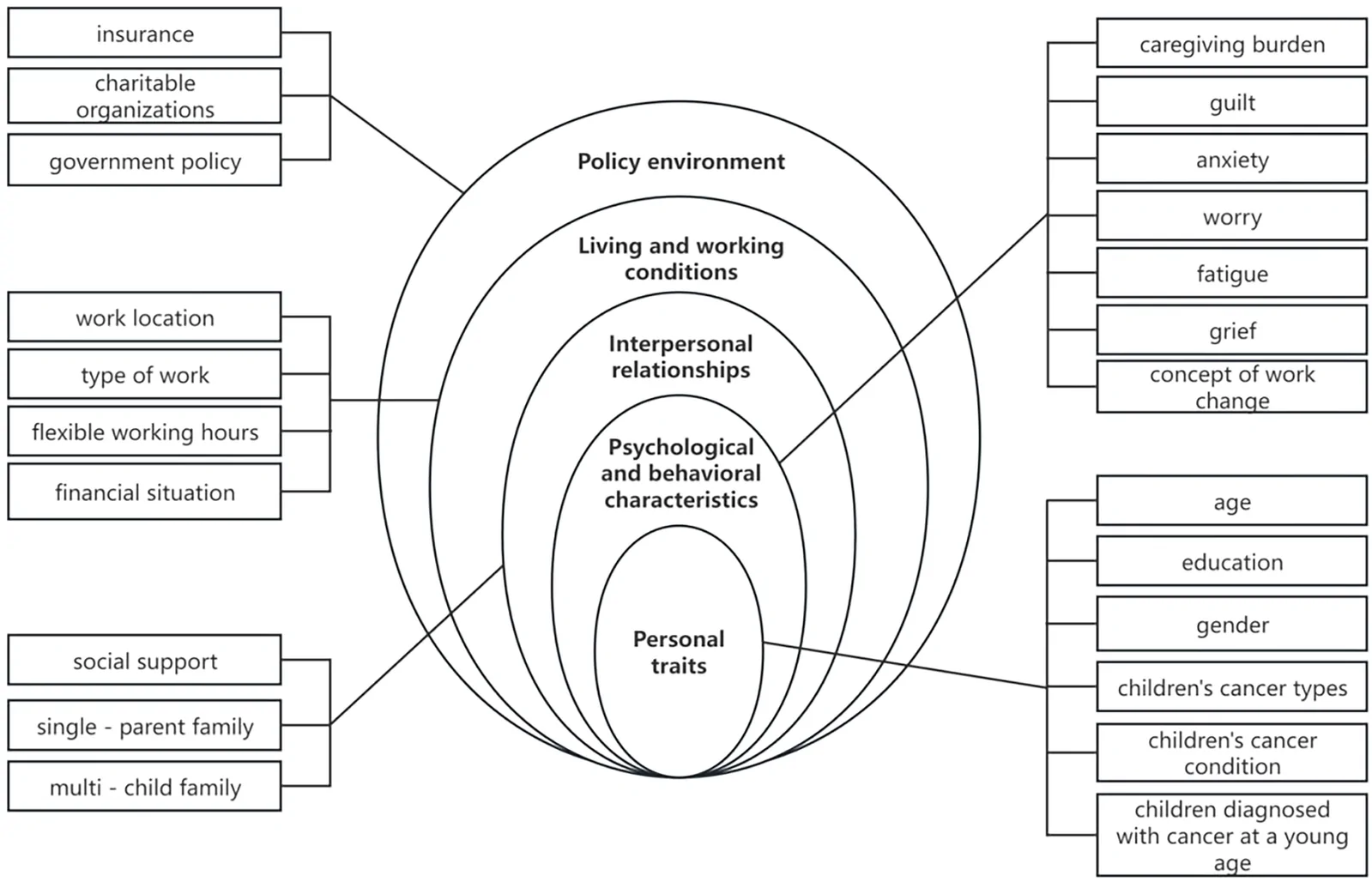
The model of the impact of minor children with cancer on parents’ work.

Personal traits: gender^(25,26,27,31,33,37,40,42)^, age^(26,28)^ , education^(26,28,37)^, children diagnosed with cancer at a young age^(26,32)^, children’s cancer condition^(39,40)^, children’s cancer types^(25,37)^;

Psychological and behavioral characteristics: guilt^(28,30)^ , anxiety^(30,41,42)^, worry^(29,31,32,41)^, fatigue^(10,31,39,41)^ , grief^(27,28,41,42)^, concept of work change^(10,30,32,34,39,43)^, caregiving burden^(10,29,30,31,32,38,39,41,42)^;

Interpersonal relationships: social support^(25,29,30,31,32,34,35,38,39,41)^, single-parent family^(28,35)^, multi-child family^(37,40,41)^;

Living and working conditions: work location^(31,32,34)^, type of work^(31,32,34)^, flexible working hours^(30,31,32)^, financial situation^(10,30,31,34,35,38,39,41)^

Policy environment: insurance^(31,34)^, charitable organizations^(25,34,35)^, government policy^(25,31,34,35)^.

### Work Adjustments Parents make after their Minor Child is Diagnosed with Cancer

The 21 included studies revealed various work impacts experienced by parents during the period between their child’s cancer diagnosis and death. These impacts can be divided into three categories: 9 studies^(10,30,32,35,38,40,41,42,43)^ measured changes in parents’ working hours, 12 studies^(10,26,28,29,30,31,32,34,36,38,40,41)^ measured parents’ resignation or job changes, and 12 studies^(10,27,28,29,30,32,33,34,35,36,38,40)^ measured parents’ leave of absence. See Table 2 for details.

## DISCUSSION

This study includes 21 research articles and demonstrates that having a child with cancer significantly and continuously impacts parents’ work. This effect is typically the result of multiple factors interacting across different levels. To address this, we used the Ecological Model of Health to systematically categorize the factors influencing parents’ work when their minor child is diagnosed with cancer. These factors include personal traits, psychological and behavioral characteristics, interpersonal relationships, living and working conditions, and policy environment. Additionally, we examined the work adjustments parents make at different stages of their child’s treatment, which can include taking sick leave, fluctuating work hours, resigning, and changing jobs.

### Categorization of Influencing Factors Based on the Ecological Model of Health

Guided by the Ecological Model of Health, we integrated various influencing factors. First, at the level of individual traits, our research found that mothers’ work is more severely affected than fathers’. Based on Swedish national registry data, Lindahl Norberg et al.^(26)^ found that after a child’s cancer diagnosis, mothers’ employment income decreased by 21% and remained affected for up to six years, whereas fathers’ income decreased by 10% and recovered after approximately three years. These differences may be attributed to traditional gender role expectations, in which mothers are expected to take primary responsibility for caregiving, and children often rely more on mothers for daily care and emotional support^(44)^. Our results are consistent with Leemann et al.^(45)^ findings, which indicate differences in the sick children’s care needs towards their parents. Additionally, factors such as parents’ age and occupation significantly influence work stability. Tamoliūnė et al.^(46)^ research found that young parents, during critical periods of work development, are prone to work stagnation or skill decline due to increased family responsibilities.

Second, at the level of psychological and behavioral characteristics, parents’ mental health and the burden of childcare are key factors affecting work. After a child is diagnosed with cancer, parents commonly experience anxiety and fatigue due to the uncertainty of the disease, such as complications and recurrence risks^(47)^. In families with multiple children, parents’ energy is often focused on the child with cancer, which can lead to guilt about neglecting the needs of other children and increase psychological burden. Existing evidence suggests that greater psychological burden is associated with poorer work performance^(48)^.

Third, from an interpersonal perspective, the financial support and emotional comfort from family and friends, as well as flexible work arrangements offered by colleagues and employers, all contribute to improving parents’ work stability. Previous research has shown that employees from different family structures have distinct needs and resources for balancing work and family responsibilities, highlighting the importance of providing tailored support for different parent groups to help them maintain work stability^(49)^. Therefore, providing personalized support to different groups becomes particularly important to help them cope with the challenges of family responsibilities and maintain work stability. Notably, workplace cultural differences can affect interpersonal interactions and thus influence work performance^(50)^. For example, in Korea, frequent social gatherings outside of work are common, but fathers with young children suffering from cancer may reduce their participation in social activities to care for their children, which may negatively impact their work performance^(30)^.

Fourth, regarding living and working conditions, parents working in large corporations or government institutions benefit from more supportive policies and flexible work arrangements, helping them balance caregiving and work development. This finding aligns with previous research^(51)^, which showed that employees in small and medium-sized enterprises have poorer work-life balance compared to those in larger organizations. In addition, socioeconomic status is an important factor influencing work changes. Low-income families not only face heavy medical costs but are also more likely to experience income loss and work stagnation due to reduced working hours^(7)^.

Finally, at the policy environment level, our research results found that parents purchasing health insurance, obtaining national welfare policies, and receiving charitable assistance play an important role in mitigating the impact of work and family role conflict caused by unexpected events. Evidence suggests that, compared with other poverty alleviation policies, critical illness insurance has a stronger poverty-reduction effect^(52)^. Therefore, focusing on low-income populations, increasing relevant national welfare policies, and improving social security mechanisms can enhance families’ economic resilience.

### Parents’ Work Adjustments at Different Stages of Their Child’s Cancer Treatment

Another key finding of this study is that parents make different work adjustments at various stages of their child’s cancer treatment, primarily involving taking sick leave, fluctuating work hours, resigning, or changing jobs. At the initial diagnosis stage, parents must quickly respond to a major family event while balancing work and caregiving responsibilities. Most parents make work adjustments, with fathers in two-parent households more likely to increase their working hours to boost income, while mothers are more likely to reduce work hours or take sick leave to care for their child. Previous research indicates that daily caregiving time for a child with cancer can reach up to 9 hours^(53)^, which exacerbates the conflict between work and family, prompting parents to adjust their work schedules to maintain balance.

The financial burden of cancer treatment often exceeds 10% of the family’s annual income^(54)^, with national health insurance systems varying in the proportion of family income spent on medical costs. Generally, expenditures exceeding 10% of annual income are considered catastrophic^(55)^. As a result, mothers are more likely to reduce work hours or take sick leave, which further increases the financial burden, while fathers tend to extend their work hours to compensate for income loss. When their children’s cancer enters the stable treatment phase or the late stage of treatment, the most parents’ work status gradually returns to a new ‘normal’. During this phase, parents typically choose to return to work, but reduce their working hours or change jobs to maintain a certain level of income to care for their children. These adjustments are mainly driven by reduced uncertainty in treatment, relatively stable child health status^(56)^, and treatment shifting to regular checkups or phased treatment, which allows parents to reallocate time between work and family.

In addition, it is worth noting that after the death of a child, parents usually do not return to work in the short term, often requiring about two years to re-enter the workforce. This delay is likely due to the immense grief of losing a child, which causes parents to lose interest in work and struggle to concentrate. A systematic review by Yaffe and Levkovich found that 45% of bereaved parents experience prolonged grief disorder^(57)^. The results of this study are consistent with previous research, indicating that grief not only has a profound impact on parents’ mental health but may also impair their cognitive functions and social skills^(58,59)^.

### Practical Implications

These findings have several theoretical and practical implications. First, further research is needed to explore the mechanisms through which pediatric cancer affects parental work status, to develop theory-based models that can inform future research and identify potential intervention targets. Second, in terms of intervention research and clinical practice, parents often experience substantial physical and psychological burdens after their minor child is diagnosed with cancer. Current interventions primarily focus on alleviating caregiving burden and psychological distress^(60,61)^, but pay less attention to the dynamic changes in parents’ needs and their struggle to balance work and caregiving. In fact, parents’ needs change significantly throughout the child’s treatment trajectory. Future intervention research should therefore focus on the specific challenges parents face at each stage of their child’s treatment and provide targeted, personalized support for maintaining employment and balancing caregiving responsibilities. For example, nurses in clinical practice currently concentrate mainly on the treatment and health outcomes of pediatric cancer patients, often neglecting the long-term psychological, financial, and occupational impacts on families^(62,63)^. In this context, nurses can assess parents’ conflicts between work and caregiving and support needs, provide individualized guidance, and coordinate multidisciplinary resources such as social work and psychological counseling to help families balance treatment, caregiving, and employment responsibilities.

From a policy perspective, existing support measures remain insufficient in terms of coverage, accessibility, and duration to fully meet the continuing needs of parents of children with cancer throughout the long-term processes of treatment, rehabilitation, and follow-up^(64)^. Access to healthcare resources and continuity of services are critical not only for children’s treatment outcomes but also for parents’ ability to maintain employment. Therefore, interdisciplinary and cross-sector collaboration should be strengthened by integrating medical, nursing, psychological, social work, employment support, and social security resources. Such efforts may improve service continuity and coordination, and help parents achieve a more sustainable balance between caregiving and work. Furthermore, across different national and cultural contexts, mothers are often expected to assume greater caregiving responsibilities, and prolonged caregiving may increase their risk of work interruption and restricted career development^(10,31,44)^. Although labor regulations and social protection systems vary across countries, measures such as paid sick leave, caregiving leave, flexible work arrangements, employment protection, health insurance, financial assistance, and charitable support can help alleviate work-family conflict and strengthen families’ economic resilience^(65,66)^. Therefore, future policies should further improve labor protection and social protection systems and develop more continuous, accessible, and equitable support mechanisms to reduce the long-term risks of financial instability, parental work disruption, and psychosocial distress.

### Limitations

This study has several limitations. First, as a scoping review, the included studies varied significantly in design, definitions of work outcomes, and follow-up periods, which limited cross study comparability and prevented quantitative synthesis. Second, possible publication bias should be acknowledged. This study included published, peer-reviewed, English-language original studies, which may have led to the omission of grey literature and studies published in other languages. Furthermore, only five databases were searched, which may have resulted in the omission of some relevant studies. Third, although 21 studies from 10 countries were included, the evidence was predominantly from high-income settings, with limited representation from low and middle-income countries. Given that cultural norms, labor laws, and social protection systems vary across countries, parental work adjustment patterns may not be directly transferable across different contexts. These limitations may constrain the generalizability of the findings and the broader applicability of the policy implications.

## CONCLUSION

This study shows that having a minor child with cancer can cause substantial and lasting disruptions to parental work. Guided by the Ecological Model of Health, we examine the factors contributing to these disruptions at multiple levels, including personal traits, psychological and behavioral characteristics, interpersonal relationships, living and working conditions, and the policy environment. Under the influence of these factors, parents often make significant adjustments to their work, such as taking extended sick leave, adjusting working hours, resigning, or changing jobs. Future intervention research should focus on the challenges parents face at different stages of their child’s treatment, offering targeted and personalized work-related interventions. Additionally, policymakers should strengthen healthcare and work-related support for parents of children with cancer, helping to alleviate their burdens and enabling them to better balance family and work.

## Data Availability

The entire dataset supporting the results of this study was published in the article itself.
