# Supplementary material for: The impact of a minor child’s cancer diagnosis on parental work: a scoping review
Source: Rev Esc Enferm USP. 2026 Oct 2;60:e20260175. doi: 10.1590/1980-220X-REEUSP-2026-0175en (PMC13638201; doi:10.1590/1980-220X-REEUSP-2026-0175en)
Supplement: Translation

# Translation: El impacto del diagnóstico de cáncer de un menor en el trabajo de los padres: revisión del alcance

**DOI:** 10.1590/1980-220X-REEUSP-2026-0175es

**Authors:** Shuyuan Shi, Yuqi Jiang, Hongyan Zhao, Wen Lu, Yuwen Zhou, Yujie Guo

**Affiliations:** 1 Nantong University, School of Nursing and Rehabilitation, Nantong, China.; 2 Taizhou Second People’s Hospital Affiliated to Yangzhou University, Taizhou, China.

**Keywords:** Niño, Neoplasias, Trabajo, Padres, Revisión de Alcance

## Abstract

**Objetivo:**

Este estudio tiene como objetivo analizar sistemáticamente el impacto de tener un hijo menor de edad con cáncer en el trabajo de los padres, incluyendo los factores que influyen y las estrategias de ajuste dinámicas.

**Método:**

El estudio utilizó el marco de Arksey y O’Malley y la lista PRISMA-ScR para guiar el proceso de revisión y minimizar el posible sesgo. El Modelo Ecológico de la Salud se adoptó como marco teórico para analizar los factores influyentes extraídos de los estudios incluidos. Realizamos búsquedas en las bases de datos PubMed, Excerpt Medica Database (Embase), Web of Science, Cumulative Index to Nursing and Allied Health Literature (CINAHL) y Cochrane Library para encontrar artículos publicados desde la fecha de creación de cada base de datos hasta el 31 de diciembre de 2025. Dos investigadores realizaron la selección de artículos y la extracción de datos.

**Resultados:**

Se recuperaron un total de 8.236 artículos, de los cuales 21 cumplían los criterios de inclusión. Guiado por el Modelo Ecológico de la Salud, este estudio demuestra que el trabajo parental está influenciado por la interacción de múltiples factores. Los ajustes laborales que deben realizar los padres son dinámicos y pueden incluir bajas por enfermedad, cambios en el horario laboral, dimisión o cambios de trabajo.

**Conclusión:**

Cuando a un niño se le diagnostica cáncer, el trabajo de los padres suele verse afectado de forma significativa. Por lo tanto, se necesitan intervenciones personalizadas en el lugar de trabajo y un mayor apoyo de políticas para ayudarles a conciliar el trabajo y las responsabilidades de cuidado.

## INTRODUCCIÓN

Con el desarrollo de las tecnologías de detección temprana y tratamiento del cáncer, han aumentado tanto la incidencia como las tasas de supervivencia de los menores con cáncer^(1)^. Según la Organización Mundial de la Salud (OMS), cada año se diagnostica cáncer a aproximadamente 400 000 niños^(2)^, y la tasa de incidencia mundial sigue aumentando^(3)^. Tras recibir un diagnóstico de cáncer, los menores suelen someterse a un ciclo de tratamiento prolongado, acompañado de efectos secundarios significativos^(4)^. Durante este proceso, los pacientes pediátricos con cáncer suelen depender en gran medida de la atención continua, el apoyo emocional y la seguridad económica que les brindan sus padres, lo que puede tener un impacto significativo, multidimensional y duradero en la vida social de estos^(5,6)^. Por ejemplo, en el ámbito económico, la carga financiera de la familia aumenta significativamente, y el 63% de las familias se enfrenta a la doble presión del aumento vertiginoso de los costos del tratamiento y la disminución de los ingresos^(7)^. En cuanto a la salud mental, el bienestar físico y emocional de los padres también se ve afectado de manera significativa, y los niveles de ansiedad y depresión aumentan notablemente durante el tratamiento^(8)^. En lo que respecta al sueño, el cuidado de hijos menores con cáncer suele consumir una gran cantidad de tiempo y energía de los padres, y el 72% de los cuidadores reporta una grave privación del sueño^(9)^.

Estas influencias multifacéticas se entrelazan y afectan colectivamente el trabajo de los padres. El diagnóstico de cáncer en un niño puede provocar interrupciones laborales para los padres, una reducción de las horas de trabajo, obstáculos en el desarrollo profesional e incluso el riesgo de abandonar por completo el mercado laboral^(10)^. Por ejemplo, los padres necesitan tomar licencia por enfermedad o reducir sus horas de trabajo para brindar atención médica continua a su hijo. Estudios previos han demostrado que la estabilidad laboral de los padres desempeña un papel crucial en el tratamiento y la recuperación de los niños con cáncer^(11,12)^. Una vez que el trabajo de los padres se ve afectado, la continuidad y la previsibilidad de los ingresos del hogar disminuyen, lo que agrava la presión financiera derivada de los gastos médicos y, por lo tanto, limita el acceso y la inversión sostenida en recursos relacionados con el tratamiento^(13)^. Al mismo tiempo, el conflicto de roles y la presión financiera derivados de la inestabilidad laboral pueden aumentar la carga psicológica de los padres, debilitar el funcionamiento familiar e interrumpir la continuidad de los cuidados. Esto puede tener efectos adversos en la salud mental de los miembros de la familia, la rehabilitación física de los niños y su calidad de vida a largo plazo^(14)^.

Sin embargo, hasta la fecha ningún estudio ha revelado de manera sistemática qué factores influyen en el trabajo de los padres cuyos hijos menores padecen cáncer, ni qué ajustes laborales realizan los padres en las diferentes etapas del tratamiento de sus hijos. La literatura existente se ha centrado principalmente en los impactos no relacionados con el trabajo, abordando sobre todo el estrés psicológico de los padres^(15)^, las cargas del cuidado^(16)^ y la toxicidad financiera^(17)^, mientras que los factores directamente relacionados con el trabajo aparecen de manera fragmentada. Además, existe una falta de diálogo coherente e integración entre los estudios respecto a los ajustes laborales específicos que realizan los padres, lo que da lugar a hallazgos dispersos y poco concluyentes. Como resultado, no se ha desarrollado evidencia integral que sirva de guía para la práctica y las políticas. Para abordar esta brecha, llevamos a cabo una revisión de alcance con el objetivo de resumir sistemáticamente la evidencia existente sobre cómo el cáncer infantil afecta el trabajo de los padres, identificar los factores que influyen en su trabajo y explorar los ajustes laborales que los padres adoptan tras el diagnóstico de cáncer de su hijo.

## MÉTODO

Dada la amplitud del tema de investigación, esta revisión de alcance se llevó a cabo de manera sistemática de acuerdo con las directrices metodológicas establecidas para este tipo de revisiones^(18)^ y se presentó siguiendo la extensión de los Elementos Preferidos de Reportaje para Revisiones Sistemáticas y Metaanálisis (PRISMA-ScR)^(19)^. Además, se empleó el marco metodológico de cinco etapas de Arksey y O’Malley para guiar el proceso de revisión y minimizar posibles sesgos^(20)^. Cabe señalar que, debido a las características de las revisiones de alcance, no se requiere una evaluación de la calidad metodológica y, por lo tanto, no se llevó a cabo para los estudios incluidos. Además, el protocolo se registró en el Centre for Open Science (doi: https://doi.org/10.17605/OSF.IO/BHSJA).

### Identificación de las Preguntas de Investigación

(1) ¿Qué factores influyen en el trabajo de los padres después de que a su hijo menor de edad se le diagnostique cáncer?
(2) ¿Qué ajustes laborales realizan los padres después de que a su hijo menor de edad se le diagnostique cáncer?

### Estrategia de Búsqueda

Realizamos una búsqueda sistemática en las bases de datos PubMed, Excerpt Medica Database (Embase), Web of Science, Cumulative Index to Nursing and Allied Health Literature (CINAHL) y la Biblioteca Cochrane para encontrar artículos publicados desde la fecha de inicio de la base de datos hasta el 31 de diciembre de 2025. La estrategia de búsqueda incorporó tanto encabezados de materia como palabras clave. En la Tabla 1 se presenta un ejemplo de la estrategia de búsqueda detallada implementada en PubMed. Se examinaron las listas de referencias de los estudios incluidos y se realizó un seguimiento de las citas para identificar estudios relevantes adicionales.

**Tabla 1:** Estrategia de búsqueda para PubMed – Multiples countries, 2000–2025.

No.	Estrategia de búsqueda	Registros, n
1	((((((“Child”[Mesh]) OR (Minor*[Title/Abstract])) OR (Adolescent*[Title/Abstract])) OR (infant*[Title/Abstract])) OR (teenager*[Title/Abstract])) OR (children[Title/Abstract])) OR (teen*[Title/Abstract])	3,238,829
2	((((“Neoplasms”[Mesh]) OR (cancer*[Title/Abstract])) OR (tumor*[Title/Abstract])) OR (neoplasm[Title/Abstract])) OR (malignan*[Title/Abstract])	5,381,295
3	((((((“Family”[Mesh]) OR “Parents”[Mesh]) OR “Caregivers”[Mesh]) OR (fami*[Title/Abstract])) OR (parent*[Title/Abstract])) OR (carer*[Title/Abstract])) OR (father[Title/Abstract])) OR (mother[Title/Abstract]) OR (family caregivers[Title/Abstract]) OR (informal caregivers[Title/Abstract])	2,159,156
4	(((((((((“Work”[Mesh]) OR (Job[Title/Abstract])) OR (Employment[Title/Abstract])) OR (Occupations[Title/Abstract])) OR (Unemployment[Title/Abstract])) OR (Income[Title/Abstract])) OR (Work Engagement[Title/Abstract])) OR (workplace[Title/Abstract])) OR (work market[Title/Abstract])) OR (Work-Life Balance[Title/Abstract])	429,894
5	#1 AND #2 AND #3 AND #4	1,181

Abreviaciones: MeSH, Medical Subject Headings. Los filtros usados fueron los siguientes: “full text,””English,””2000/01/01–2025/12/31.”

### Criterios de Elegibilidad

La pregunta de investigación se definió utilizando el principio PCC (Población, Concepto, Contexto)^(21)^: (1) Población: padres de niños menores de edad diagnosticados con cáncer; (2) Concepto: el impacto de los niños menores de edad con cáncer en el trabajo de los padres, incluyendo los factores que influyen y las estrategias dinámicas de adaptación; (3) Contexto: estudios de diversos países y entornos de atención médica, considerando diferentes situaciones socioeconómicas, entornos laborales y contextos culturales.

### Criterios de Inclusión y Exclusión

Criterios de inclusión: (1) Padres de niños y adolescentes diagnosticados con cáncer ≤18 años; (2) Contenido de la investigación que aborde explícitamente el impacto de la situación laboral de los padres de menores con cáncer; (3) Diseño de estudio que incluya investigación original con métodos cualitativos, cuantitativos o mixtos (incluidos estudios longitudinales o transversales); (4) Idioma de la publicación: inglés; (5) Publicados en revistas revisadas por pares entre 2000 y 2025.

Criterios de exclusión: (1) Resúmenes de congresos; (2) Estudios que describieran únicamente los impactos psicológicos y de salud en los padres de niños con cáncer sin abordar las consecuencias relacionadas con el trabajo; (3) Estudios cuyo texto completo no se pudiera obtener a través de enlaces a bases de datos, acceso institucional, sitios web de revistas, verificación de referencias y búsquedas de texto completo en línea.

### Selección de Estudios y Extracción de Datos

Todos los registros bibliográficos recuperados se cargaron en Endnote X9 para la gestión de la bibliografía y la identificación de duplicados, los cuales se eliminaron antes de la selección. Los registros que debían someterse a selección se importaron a Rayyan (https://www.rayyan.ai/) para realizar una selección ciega de títulos y resúmenes. Dos revisores evaluaron de manera independiente todos los títulos y resúmenes según los criterios de elegibilidad predefinidos. En caso de desacuerdo, consultaban y discutían con un tercer investigador hasta alcanzar un consenso. Además, se revisaron manualmente las listas de referencias de los estudios incluidos para identificar otras publicaciones elegibles. La recolección de datos fue realizada de manera independiente por dos revisores utilizando un formulario de extracción de datos prediseñado. Los datos recolectados incluyeron el autor principal, el año de publicación, el país, el diseño del estudio, el tamaño de la muestra, los ajustes laborales de los padres, el estado clínico del niño y los factores que influyen en el trabajo de los padres.

Para organizar y sintetizar sistemáticamente los factores que afectan el trabajo de los padres, se adoptó el Modelo Ecológico de la Salud como marco analítico, y los factores identificados se clasificaron en múltiples niveles. Este modelo, propuesto en la investigación sobre promoción de la salud, postula que los resultados relacionados con la salud y los cambios de comportamiento no están determinados únicamente por las características individuales, sino más bien por los efectos combinados de múltiples factores relacionados con el individuo y su entorno social^(22)^. Este modelo se adopta principalmente por dos razones. En primer lugar, se utiliza ampliamente en la investigación relacionada con la salud y el cáncer, orientando las intervenciones multinivel, particularmente en la atención oncológica, y haciendo hincapié en el apoyo sistémico desde el nivel individual hasta el de las políticas^(23,24)^. En segundo lugar, el modelo proporciona una clasificación jerárquica clara y una lógica de evaluación, lo que ayuda a incorporar factores relacionados con el trabajo — que antes estaban dispersos—en un sistema de clasificación unificado, formando así un panorama estructurado, integral y comparable de la evidencia. Todos los resultados de la extracción y clasificación de datos fueron discutidos por el equipo de investigación y finalizados por consenso.

## RESULTADOS

### Información General de la Bibliografía Incluida

Se identificaron un total de 8236 artículos en la búsqueda inicial. Tras eliminar 1601 duplicados, se revisaron los títulos y resúmenes de 6635 artículos, lo que dio lugar a la exclusión de 6553 artículos. Los 82 artículos restantes se evaluaron de acuerdo con los criterios de elegibilidad. Finalmente, se incluyeron en la revisión 21 artículos elegibles. El proceso detallado de selección de estudios se muestra en la Figura 1.

**Figura 1:** Diagrama de flujo de la selección de la literature.

Los estudios incluidos se llevaron a cabo en diez países: Estados Unidos, Australia, Alemania, Suecia, Suiza, Dinamarca, Japón, Corea del Sur, Canadá y Finlandia. De los 21 estudios, cinco fueron estudios de cohorte retrospectivos, tres fueron estudios de cohorte prospectivos, cinco fueron estudios transversales, siete fueron estudios cualitativos y uno fue un análisis de casos retrospectivo. Estos estudios se publicaron entre 2004 y 2025 y se centraron en padres de niños de 0 a 18 años a quienes se les había diagnosticado cáncer. Los períodos analizados abarcaron desde el momento del diagnóstico, pasando por el proceso de tratamiento, el final de la fase de tratamiento y el período posterior al fallecimiento del niño, con el fin de explorar el impacto de estas diferentes etapas en el trabajo de los padres.

El tamaño total de la muestra de esta revisión de alcance fue de 14 009 padres de niños con cáncer. Entre los estudios incluidos, el de Goodenough et al.^(25)^ solo informó el número de familias, no de padres individuales, por lo que se excluyó del cálculo del tamaño total de la muestra. Además, los estudios de Lindahl Norberg^(26)^ y Hjelmstedt^(27)^ utilizaron datos del Registro Sueco de Cáncer Infantil (SCCR), que incluía al mismo grupo de participantes (n = 3 626). Para evitar el doble conteo, esta muestra se incluyó solo una vez en el total general. La información básica de la bibliografía incluida se muestra en la Tabla 2.

**Tabla 2:** Información general de los estudios incluidos – Multiples countries, 2000–2025.

Autores y país	Tipos de artículos	Características de los padres de niños con cáncer	Características de los niños con cáncer	Descripción de los resultados
Ospelt et al.^(10)^ Suiza	Estudio transversal	276 madres (M ± DE = 37,3 ± 5,9 años, rango de 21 a 57 años) 193 padres (media ± desviación estándar = 39,9 ± 6,5 años, rango de 23 a 64 años)	Edad en el momento del diagnóstico: ≤18 años. Habían transcurrido al menos 5 años desde el diagnóstico.	267 madres: 70 informaron de cambios en su situación laboral, incluidas 33 que redujeron su jornada laboral, 23 que dimitieron, 13 que se acogieron a un permiso sin sueldo, 3 que cambiaron de trabajo y 1 que aumentó su jornada laboral. 193 padres: 28 declararon cambios en su situación laboral, entre ellos 17 que redujeron su jornada laboral, 3 que dimitieron, 7 que se acogieron a un permiso no remunerado, 2 que cambiaron de trabajo, 2 que aumentaron su jornada laboral y 1 que quedó en paro.
Hammer et al.^(28)^ Dinamarca	Estudio de cohorte retrospectivo	1.609 padres cuyo hijo falleció de cáncer (madres: n = 857, mediana [IQR] = 37,5 [33,2 – 42,4] años, rango 19,8-60,3 años), (padres: n = 752, mediana [IQR] = 39,7 [34,8 – 45,6] años, rango 22,9–63,8 años).	Edad en el momento del diagnóstico: ≤18 años. Fallecimiento antes de los 30 años (mediana = 8,3 años). Desde un año antes de la muerte del hijo o de la fecha de referencia hasta cinco años después.	Madres que han perdido a un hijo por cáncer: Aumento general del riesgo de baja por enfermedad de larga duración (HR = 1,62; IC del 95%: 1,48–1,77), observándose el efecto más marcado en los dos primeros años tras la muerte del hijo (HR = 3,74; IC del 95%: 3,25–4,31). Menor probabilidad de trabajar durante los dos primeros años tras la pérdida (OR = 0,44; IC del 95%: 0,39–0,49), con una media de 15 meses de trabajo en los dos primeros años, frente a los 18 meses de las madres no expuestas. Mayor riesgo de desempleo (HR = 1,53; IC del 95%: 1,37–1,70), con el efecto más marcado en los dos primeros años tras la pérdida (HR = 1,95; IC del 95%: 1,69–2,26). Padres que han perdido a un hijo por cáncer: No se observó un aumento general del riesgo de baja por enfermedad, pero sí un mayor riesgo de baja por enfermedad de larga duración en los dos primeros años tras la pérdida (HR = 2,10; IC del 95%: 1,67–2,63), y un riesgo ligeramente menor tras cinco años (HR = 0,81; IC del 95%: 0,67–0,96). Menor probabilidad de trabajar (OR = 0,65; IC del 95%: 0,53–0,79), con una media de 21 meses de trabajo frente a los 22 meses de los padres no expuestos. Mayor riesgo de sufrir una reducción permanente de la capacidad laboral (HR = 1,29; IC del 95%: 1,01–1,66).
Okada et al.^(29)^ Japón	Estudio transversal	171 madres (mediana [IQR] = 44 [41-49] años)	Edad en el momento del diagnóstico: (mediana [IQR] = 4 [1-8] años). Edad en el momento del diagnóstico: ≤15 años. Tiempo transcurrido desde que finalizó el tratamiento oncológico de sus hijos: (media [IQR] = 60 [24–114] meses).	En el momento del diagnóstico, el 59,6% de las madres seguían trabajando. De ellas, el 40,0% de las empleadas fijas y el 18,6% de las empleadas temporales solicitaron una baja prolongada, mientras que el 36,0% de las empleadas fijas y el 58,1% de las empleadas temporales dejaron sus puestos de trabajo. En el momento de la encuesta, el 75,4% de las madres tenían empleo. De ellas, el 48,5% eran trabajadoras con contrato temporal, el 21,6% tenían contrato indefinido y el 5,3% trabajaban por cuenta propia.
Yi et al.^(30)^ Corea	Estudio cualitativo	20 padres (M ± DE = 41,35 ± 4,49 años, rango: 32–49 años)	Edad en el momento del diagnóstico: (M ± DE = 6,35 ± 4,08 años, rango = 1–16 años). En los 5 años siguientes al diagnóstico.	En las primeras fases del tratamiento: Las madres tienen que cuidar de sus hijos las 24 horas del día, mientras que los padres tienen que compaginarlo con otros trabajos. Los padres salen antes del trabajo para pasar más tiempo con sus hijos.
Hjelmstedt et al.^(31)^ Suecia	Estudio cualitativo	21 madres y 11 padres (mediana = 44 años, rango: 31–51 años)	Edad en el momento del diagnóstico: ≤18 años. Años transcurridos desde el diagnóstico (mediana = 5 años, rango = 1–16 años).	Los padres preferirían dedicar toda su energía a sus hijos, pero para gestionar las finanzas familiares se vieron obligados a seguir trabajando o a reincorporarse al trabajo lo antes posible. Algunos padres y madres afirmaron que, debido a que estaban demasiado centrados en el cuidado de sus hijos, fueron despedidos por sus empleadores, sufrieron retrasos en el pago de sus salarios e incluso vieron estancadas sus carreras profesionales.
Peikert et al.^(32)^ Alemania	Estudio cualitativo	29 madres (M ± DE = 42 ± 7,9 años, rango = 26–65 años) 20 padres (M ± DE = 43,4 ± 5,9 años, rango = 31–52 años)	Edad en el momento del diagnóstico: (M ± DE = 5,5 ± 4,5 años, rango = 0-17 años). Entre 16 y 24 meses después de un tratamiento intensivo contra el cáncer.	En más de la mitad de las familias, la enfermedad del niño provocó que al menos uno de los padres se acogiera a una baja por enfermedad durante el tratamiento. El 35% de los padres redujo su jornada laboral, dejó de trabajar o eligió una profesión con mejores condiciones laborales. Dos padres perdieron su empleo como consecuencia de ello.
Hjelmstedt et al.^(33)^ Suecia	Estudio de cohorte retrospectivo	1 394 madres (M ± DE = 36,75 ± 6,89 años) 1 394 padres (M ± DE = 39,36 ± 7,44 años)	Edad en el momento del diagnóstico: ≤18 años. Desde un año antes del diagnóstico hasta siete años después del mismo.	Cuando a un niño se le diagnostica cáncer, el TPL (permiso parental temporal) de los padres aumenta drásticamente. En el plazo de un año desde el diagnóstico de su hijo, las madres y los padres de niños con cáncer tuvieron una TPL media de 48,3 días y 32,2 días, respectivamente, en comparación con una TPL media de 3,3 días y 2,6 días para las madres y los padres sin cáncer, respectivamente. Cinco años después del diagnóstico, las madres con hijos con cáncer seguían teniendo tasas de TPL significativamente más altas que los padres sin hijos con cáncer, mientras que los padres con hijos con cáncer seguían teniendo tasas de TPL significativamente más altas seis años después del diagnóstico. Entre los padres de niños con cáncer, las madres tuvieron más días de TPL que los padres desde un año antes del diagnóstico hasta cinco años después del mismo.
Kelada et al.^(34)^ Australia	Estudio cualitativo	50 madres y 6 padres (M ± DE = 40,21 ± 6,3 años, rango = 20–55 años)	Edad en el momento del diagnóstico: (M ± DE = 5,17 ± 4,72 años, rango = 0-18 años). Años transcurridos desde el diagnóstico (M ± DE = 2,89 ± 2,26 años). Años transcurridos desde la finalización del tratamiento (M ± DE = 2,13 ± 2,04 años).	Veintinueve padres (51,8%) indicaron que ellos y/o sus parejas redujeron su jornada laboral o dejaron su trabajo tras el diagnóstico de su hijo. Las madres parecían más propensas que los padres a dejar de trabajar por completo. Los participantes y sus parejas redujeron sus horas de trabajo tomando permisos no remunerados (13, 23,2%), tomando permisos remunerados prolongados (incluidas las bajas por enfermedad y las bajas por antigüedad) (5, 8,9%), pasando de un trabajo a tiempo completo a uno a tiempo parcial o eventual (6, 10,7%), modificando el horario de trabajo en el caso del trabajo por cuenta propia (3, 5,4%), dejando su trabajo (12, 21,4%) o una combinación de estas opciones.
Tsimicalis et al.^(35)^ Canadá	Estudio cualitativo	78 padres de niños con cáncer (madres: M ± DE = 37,83 ± 7,95 años), (padres: M ± DE = 40,31 ± 7,60 años)	Edad en el momento del diagnóstico: (M ± DE = 7,72 ± 5,4 años, rango = 0–18 años). Diagnosticados con cáncer en los 21 días anteriores.	Uno de los padres o ambos aumentan sus horas de trabajo remunerado para compensar la pérdida de ingresos.
Hjelmstedt et al.^(27)^ Suecia	Estudio de cohorte retrospectivo	1.881 madres (media ± desviación estándar = 36,8 ± 7 años) 1 745 padres (M ± DE = 39,5 ± 7,6 años)	Edad en el momento del diagnóstico: ≤18 años. Desde un año antes del diagnóstico hasta siete años después del mismo.	El riesgo de baja por enfermedad fue estadísticamente significativo hasta seis años después del diagnóstico de cáncer del niño. El aumento del número de días con prestación por enfermedad fue más pronunciado el año posterior al diagnóstico. El duelo se asoció con un mayor riesgo de baja por enfermedad, especialmente durante el año de la muerte del niño.
Lindahl Norberg et al.^(26)^ Suecia	Estudio de cohorte retrospectivo	1.881 madres (M ± DE = 36,8 ± 7 años) 1 745 padres (media ± desviación estándar = 39,5 ± 7,6 años)	Edad en el momento del diagnóstico: ≤18 años. Desde un año antes del diagnóstico hasta siete años después del mismo.	En comparación con las madres del grupo de control, las madres tenían menos probabilidades de seguir trabajando después de que a sus hijos se les diagnosticara cáncer. Este efecto fue significativo tanto en el año del diagnóstico como hasta cinco años después.
Wikman et al.^(36)^ Suecia	Estudio de cohorte prospectivo	152 padres de niños con cáncer, entre ellos 77 madres y 75 padres. (M ± DE = 38,88 ± 6,35 años) 42 padres que habían perdido a un hijo con cáncer, de los cuales 22 eran madres y 20, xml-ph-0000@deepl.internal, padres. (M ± DE = 37,19 ± 7,48 años).	Edad en el momento del diagnóstico: ≤18 años. Niños con cáncer que han recibido tratamiento contra el cáncer en los últimos 5 años .	En T7, no se observaron diferencias en la proporción de madres y padres supervivientes que trabajaban o estaban de baja por enfermedad (92% frente a 96% trabajando, 20% frente a 18% de baja por enfermedad), ni entre las madres y los padres que habían perdido a su hijo (91% frente a 90% trabajando, 14% frente a 20% de baja por enfermedad). La proporción de padres que trabajaban no varió de T6 a T7 (91% frente a 96% de los padres supervivientes, 95% frente a 90% de los padres que habían perdido a su hijo). La proporción de madres que trabajaban fue mayor entre las madres supervivientes (92% frente a 82%) y las madres que habían perdido a su hijo (91% frente a 77%) en T7 en comparación con T6; este aumento no fue significativo. La proporción de madres que habían perdido a su pareja y que se acogieron a una baja por enfermedad fue menor en T7 que en T6 (14% frente a 59%, p < 0,05). Un mayor número de padres declaró haber tomado baja por enfermedad en T7 que en T6 (18% frente a 8% en el caso de los padres supervivientes y 20% frente a 15% en el de los padres que habían perdido a su hijo), aunque esta diferencia no fue significativa.
Mader et al.^(37)^ Suiza	Estudio transversal	379 madres (M ± DE = 42,7 ± 4,8 años) 378 padres (M ± DE = 45,8 ± 5,8 años)	Edad en el momento del diagnóstico: (M ± DE = 3,1 ± 2,5 años). Tiempo transcurrido desde el diagnóstico: (M ± DE = 9,0 ± 2,5 años).	Las madres de niños con cáncer presentan una mayor tasa de desempleo, mientras que los padres tienen una mayor tasa de empleo a tiempo completo.
Lindahl Norberg et al.^(26)^ Suecia	Estudio de cohorte retrospectivo	1.881 madres (M ± DE = 36,8 ± 7 años) 1.745 padres (M ± DE = 39,5 ± 7,6 años)	Edad en el momento del diagnóstico: ≤18 años. Desde un año antes del diagnóstico hasta siete años después del diagnóstico.	Las madres eran menos propensas a seguir trabajando tras el diagnóstico de cáncer de sus hijos que las madres del grupo de control. Este efecto fue evidente durante el año del diagnóstico y hasta cinco años después del mismo. Los ingresos de los padres disminuyeron significativamente tras el diagnóstico de cáncer de sus hijos. Este efecto fue más pronunciado en las madres, cuyos ingresos continuaron disminuyendo durante seis años tras el diagnóstico, mientras que los ingresos de los padres alcanzaron la paridad con los del grupo de control en los tres años siguientes al diagnóstico.
Goodenough et al.^(25)^ Australia	Análisis retrospectivo de historias clínicas	104 familias	Edad en el momento del diagnóstico: (media = 7 años). De 2 semanas a 2 años tras el diagnóstico de cáncer. Tiempo transcurrido desde el diagnóstico: (media = 584 días).	En la muestra de 104 familias, el 58% experimentó cambios negativos en el empleo remunerado de los padres, con una pérdida media del 53% de los ingresos familiares. Alrededor del 80% de las madres trabajadoras dejaron de trabajar o redujeron su jornada laboral, mientras que solo un tercio de los padres trabajadores dejaron de trabajar o redujeron su jornada, y el pico de estos cambios se produjo en las seis semanas siguientes al diagnóstico de cáncer.
Okada et al.^(38)^ Japón	Estudio transversal	60 madres	Edad en el momento del diagnóstico: (M ± DE = 4,9 ± 4,2 años). Tiempo transcurrido desde el diagnóstico: (M ± DE = 4,4 ± 2,8 años).	De las 32 madres que trabajaban en el momento del diagnóstico, 10 continuaron trabajando, 12 se tomaron una baja prolongada y 10 dejaron de trabajar; además, el 70% perdió la motivación para trabajar tras el diagnóstico.
Wakefield et al.^(39)^ Australia	Estudio cualitativo	44 madres (M ± DE = 42,5 ± 6,7 años, rango de 29 a 64 años) 34 padres (M ± DE = 45,9 ± 8,5 años, rango de 31 a 65 años)	Edad en el momento del diagnóstico: (M ± DE = 13,3 ± 2,6 años, rango de 7 a 17 años). En los cinco años posteriores al diagnóstico y a la finalización del tratamiento.	En comparación con la situación anterior al diagnóstico de cáncer de sus hijos, los padres suelen estar dispuestos a reducir su jornada laboral y aceptar salarios más bajos a cambio de pasar más tiempo con sus familias.
Hovén et al.^(40)^ Suecia	Estudio de cohorte prospectivo	139 madres (media ± desviación estándar = 37,2 ± 6,6 años) 138 padres (M ± DE = 39,8 ± 6,8 años)	Edad en el momento del estudio: (M ± DE = 8,2 ± 5,2 años, rango 0–18 años). Desde una semana después del diagnóstico hasta un año después de finalizar el tratamiento.	Las tasas más elevadas de desempleo y bajas por enfermedad de los padres se registraron durante el periodo en que sus hijos recibían tratamiento. En comparación con el momento del diagnóstico, el número de madres que seguían trabajando tres meses después del tratamiento disminuyó, mientras que el número de madres que se acogieron a bajas por enfermedad aumentó un año después del tratamiento.
Fletcher et al.^(41)^ Canadá	Estudio cualitativo	9 madres (entre 32 y 47 años)	Edad en el momento del diagnóstico: entre 6 meses y 17 años. Niños menores de 17 años que habían recibido tratamiento contra el cáncer en los últimos 5 años; solo un niño estaba recibiendo tratamiento en el momento del estudio y cuatro habían fallecido.	Tres mujeres tuvieron que adaptar sus horarios de trabajo; tres tuvieron que tomarse una baja sin sueldo; una mujer estaba de baja por maternidad cuando le diagnosticaron la enfermedad, pero fue despedida por no reincorporarse al trabajo una vez finalizada la baja; y otra mujer tuvo que dimitir. El marido de una mujer dejó su trabajo para cuidar de los hijos.
Bennett Murphy et al.^(42)^ Estados Unidos	Estudio transversal	20 madres y 20 padres	Edad en el momento del diagnóstico: entre 18 meses y 18 años. Los niños con cáncer llevaban al menos 4 meses desde el diagnóstico.	De las 20 madres, 8 seguían trabajando. Todos los padres de los niños con cáncer trabajaban una media de 48 horas a la semana y dedicaban 4,5 horas al día al cuidado de sus hijos. (Los padres del grupo de control trabajaban una media de 42,5 horas a la semana y pasaban 4 horas al día con sus hijos)
Lähteenmäki et al.^(43)^ Finlandia	Estudio de cohorte prospectivo	24 madres (rango = 25–49 años) 19 padres (rango = 32–48 años)	Edad en el momento del diagnóstico: (media = 4,5 años, rango de 0,1 a 15 años). El primer año tras el diagnóstico de cáncer de su hijo.	En los primeros meses tras el diagnóstico de cáncer de su hijo, disminuyó la proporción de madres con hijos que trabajaban fuera de casa. Tres meses después del diagnóstico, el 33% de las madres afirmaron que habían dejado de trabajar únicamente a causa de la enfermedad de su hijo.

Nota:

T6 (un año después de finalizar con éxito el tratamiento o 18 meses después del trasplante o del fallecimiento del niño)

T7 (cinco años tras la finalización del tratamiento con éxito o el fallecimiento del niño)

TPL (permiso parental temporal)

### Factores que Influyen en el Trabajo de los Padres Tras el Diagnóstico de Cáncer de su HIJO

Hasta la fecha, ningún estudio ha resumido ni categorizado los factores que influyen en el trabajo de los padres tras el diagnóstico de cáncer de un menor. Para abordar este tema, este estudio presenta el Modelo Ecológico de la Salud como marco analítico. Basándose en este marco, esta revisión clasifica el impacto del diagnóstico de cáncer en un menor sobre el trabajo de los padres en cinco niveles: rasgos personales, características psicológicas y de comportamiento, relaciones interpersonales, condiciones de vida y de trabajo, y entorno normativo. Los factores relevantes identificados en la literatura se muestran en la Figura 2.

**Figura 2:** Modelo del impacto de los hijos menores con cáncer en el trabajo de los padres.

Rasgos personales: género^(25,26,27,31,33,37,40,42)^, edad^(26,28)^, nivel educativo^(26,28,37)^, niños diagnosticados con cáncer a una edad temprana^(26,32)^, estado del cáncer del niño^(39,40)^, tipos de cáncer del niño^(25,37)^;

Características psicológicas y de comportamiento: culpa^(28,30)^, ansiedad^(30,41,42)^, preocupación^(29,31,32,41)^, fatiga^(10,31,39,41)^, duelo^(27,28,41,42)^, cambio en la concepción del trabajo^(10,30,32,34,39,43)^, carga del cuidado^(10,29,30,31,32,38,39,41,42)^;

Relaciones interpersonales: apoyo social^(25,29,30,31,32,34,35,38,39,41)^, familia monoparental^(28,35)^, familia con varios hijos^(37,40,41)^;

Condiciones de vida y de trabajo: lugar de trabajo^(31,32,34)^, tipo de trabajo^(31,32,34)^, horarios de trabajo flexibles^(30,31,32)^, situación financiera^(10,30,31,34,35,38,39,41)^;

Entorno normativo: seguros^(31,34)^, organizaciones benéficas^(25,34,35)^, políticas gubernamentales^(25,31,34,35)^.

### Ajustes Laborales Que Realizan los Padres Después de Que a su Hijo Menor de Edad se le Diagnostica Cáncer

Los 21 estudios incluidos revelaron diversos impactos laborales que experimentaron los padres durante el período comprendido entre el diagnóstico de cáncer de su hijo y su fallecimiento. Estos impactos pueden dividirse en tres categorías: 9 estudios^(10,30,32,35,38,40,41,42,43)^ midieron los cambios en las horas de trabajo de los padres; 12 estudios^(10,26,28,29,30,31,32,34,36,38,40,41)^ evaluaron la renuncia o los cambios de empleo de los padres, y 12 estudios^(10,27,28,29,30,32,33,34,35,36,38,40)^ evaluaron las licencias laborales de los padres. Consulte la Tabla 2 para obtener más detalles.

## DISCUSIÓN

Este estudio incluye 21 artículos de investigación y demuestra que tener un hijo con cáncer tiene un impacto significativo y continuo en el trabajo de los padres. Este efecto suele ser el resultado de la interacción de múltiples factores en diferentes niveles. Para abordar esto, utilizamos el Modelo Ecológico de la Salud para categorizar sistemáticamente los factores que influyen en el trabajo de los padres cuando a su hijo menor de edad se le diagnostica cáncer. Estos factores incluyen rasgos personales, características psicológicas y de comportamiento, relaciones interpersonales, condiciones de vida y de trabajo, y el entorno normativo. Además, examinamos los ajustes laborales que realizan los padres en diferentes etapas del tratamiento de su hijo, los cuales pueden incluir tomar licencia por enfermedad, horarios de trabajo variables, renunciar al empleo y cambiar de trabajo.

### Clasificación de los Factores Influyentes Sasada en el Modelo Ecológico de la Salud

Guiados por el Modelo Ecológico de la Salud, integramos diversos factores influyentes. En primer lugar, a nivel de los rasgos individuales, nuestra investigación reveló que el trabajo de las madres se ve más gravemente afectado que el de los padres. Con base en datos del registro nacional sueco, Lindahl Norberg et al.^(26)^ encontraron que, tras el diagnóstico de cáncer de un niño, los ingresos laborales de las madres disminuyeron un 21% y se mantuvieron afectados hasta por seis años, mientras que los ingresos de los padres disminuyeron un 10% y se recuperaron después de aproximadamente tres años. Estas diferencias pueden atribuirse a las expectativas tradicionales sobre los roles de género, según las cuales se espera que las madres asuman la responsabilidad principal del cuidado, y los niños suelen depender más de las madres para el cuidado diario y el apoyo emocional^(44)^. Nuestros resultados concuerdan con los hallazgos de Leemann et al.^(45)^, que indican diferencias en las necesidades de cuidado de los niños enfermos con respecto a sus padres. Además, factores como la edad y la ocupación de los padres influyen significativamente en la estabilidad laboral. La investigación de Tamoliūnė et al.^(46)^ reveló que los padres jóvenes, durante períodos críticos de desarrollo profesional, son propensos al estancamiento laboral o a la pérdida de habilidades debido al aumento de las responsabilidades familiares.

En segundo lugar, en cuanto a las características psicológicas y de comportamiento, la salud mental de los padres y la carga que supone el cuidado de los hijos son factores clave que afectan el trabajo. Después de que a un niño se le diagnostica cáncer, los padres suelen experimentar ansiedad y fatiga debido a la incertidumbre de la enfermedad, como las complicaciones y los riesgos de recurrencia^(47)^. En las familias con varios hijos, la energía de los padres a menudo se centra en el niño con cáncer, lo que puede generar culpa por descuidar las necesidades de los demás hijos y aumentar la carga psicológica. La evidencia existente sugiere que una mayor carga psicológica se asocia con un peor desempeño laboral^(48)^.

En tercer lugar, desde una perspectiva interpersonal, el apoyo financiero y el consuelo emocional de familiares y amigos, así como los arreglos laborales flexibles que ofrecen los colegas y los empleadores, contribuyen a mejorar la estabilidad laboral de los padres. Investigaciones previas han demostrado que los empleados de diferentes estructuras familiares tienen necesidades y recursos distintos para equilibrar las responsabilidades laborales y familiares, lo que resalta la importancia de brindar apoyo personalizado a los diferentes grupos de padres para ayudarlos a mantener la estabilidad laboral^(49)^.

Por lo tanto, brindar apoyo personalizado a los distintos grupos cobra especial importancia para ayudarlos a enfrentar los desafíos de las responsabilidades familiares y mantener la estabilidad laboral. Cabe destacar que las diferencias culturales en el lugar de trabajo pueden afectar las interacciones interpersonales y, por lo tanto, influir en el desempeño laboral^(50)^. Por ejemplo, en Corea es común asistir a reuniones sociales frecuentes fuera del trabajo, pero los padres con hijos pequeños que padecen cáncer pueden reducir su participación en actividades sociales para cuidar a sus hijos, lo que podría afectar negativamente su desempeño laboral^(30)^.

En cuarto lugar, en cuanto a las condiciones de vida y de trabajo, los padres que trabajan en grandes corporaciones o instituciones gubernamentales se benefician de políticas más favorables y de modalidades de trabajo flexibles, lo que les ayuda a equilibrar el cuidado de los hijos y el desarrollo profesional. Este hallazgo concuerda con investigaciones previas^(51)^, que demostraron que los empleados de pequeñas y medianas empresas tienen un peor equilibrio entre la vida laboral y personal en comparación con los de organizaciones más grandes. Además, el estatus socioeconómico es un factor importante que influye en los cambios laborales. Las familias de bajos ingresos no solo enfrentan elevados costos médicos, sino que también son más propensas a sufrir pérdidas de ingresos y estancamiento laboral debido a la reducción de las horas de trabajo^(7)^.

Por último, en el ámbito de las políticas públicas, los resultados de nuestra investigación revelaron que el hecho de que los padres adquieran un seguro de salud, accedan a las políticas nacionales de bienestar social y reciban asistencia caritativa desempeña un papel importante en la mitigación del impacto del conflicto entre el trabajo y las responsabilidades familiares causado por eventos inesperados. La evidencia sugiere que, en comparación con otras políticas de alivio de la pobreza, el seguro contra enfermedades graves tiene un efecto más fuerte en la reducción de la pobreza^(52)^. Por lo tanto, centrarse en las poblaciones de bajos ingresos, aumentar las políticas nacionales de bienestar social pertinentes y mejorar los mecanismos de seguridad social puede fortalecer la resiliencia económica de las familias.

### Ajustes Laborales de los Padres en Las Diferentes Etapas del Tratamiento Contra el Cáncer de su Hijo

Otro hallazgo clave de este estudio es que los padres realizan diferentes ajustes laborales en las diversas etapas del tratamiento contra el cáncer de su hijo, lo que implica principalmente tomar licencia por enfermedad, modificar el horario de trabajo, renunciar o cambiar de empleo. En la etapa inicial del diagnóstico, los padres deben responder rápidamente a un evento familiar de gran importancia, al tiempo que equilibran las responsabilidades laborales y de cuidado. La mayoría de los padres realizan ajustes laborales; los padres en hogares con dos adultos tienden más a aumentar sus horas de trabajo para aumentar los ingresos, mientras que las madres tienden más a reducir sus horas de trabajo o a tomar licencia por enfermedad para cuidar a su hijo. Investigaciones previas indican que el tiempo diario dedicado al cuidado de un niño con cáncer puede llegar a ser de hasta 9 horas^(53)^, lo que agrava el conflicto entre el trabajo y la familia, lo que lleva a los padres a ajustar sus horarios laborales para mantener el equilibrio.

La carga financiera del tratamiento contra el cáncer a menudo supera el 10% de los ingresos anuales de la familia^(54)^, y los sistemas nacionales de seguro médico varían en cuanto a la proporción de los ingresos familiares que se destina a gastos médicos. En general, los gastos que superan el 10% de los ingresos anuales se consideran catastróficos^(55)^. Como resultado, las madres tienden más a reducir sus horas de trabajo o a tomar licencia por enfermedad, lo que aumenta aún más la carga financiera, mientras que los padres tienden a extender sus horas de trabajo para compensar la pérdida de ingresos. Cuando el cáncer de sus hijos entra en la fase de tratamiento estable o en la etapa final del tratamiento, la situación laboral de la mayoría de los padres vuelve gradualmente a una nueva «normalidad». Durante esta fase, los padres suelen optar por regresar al trabajo, pero reducen sus horas de trabajo o cambian de empleo para mantener un cierto nivel de ingresos que les permita cuidar a sus hijos. Estos ajustes se deben principalmente a la menor incertidumbre en el tratamiento, al estado de salud relativamente estable del niño^(56)^ y a que el tratamiento pasa a consistir en chequeos regulares o tratamientos por etapas, lo que permite a los padres redistribuir su tiempo entre el trabajo y la familia.

Además, cabe señalar que, tras la muerte de un hijo, los padres por lo general no regresan al trabajo a corto plazo, y a menudo necesitan alrededor de dos años para reincorporarse al mercado laboral. Es probable que este retraso se deba al inmenso dolor que supone la pérdida de un hijo, lo que hace que los padres pierdan interés en el trabajo y tengan dificultades para concentrarse. Una revisión sistemática realizada por Yaffe y Levkovich reveló que el 45% de los padres en duelo padecen un trastorno de duelo prolongado^(57)^. Los resultados de este estudio concuerdan con investigaciones previas, lo que indica que el duelo no solo tiene un profundo impacto en la salud mental de los padres, sino que también puede afectar sus funciones cognitivas y habilidades sociales^(58,59)^.

### Implicaciones Prácticas

Estos hallazgos tienen varias implicaciones teóricas y prácticas. En primer lugar, se necesita más investigación para explorar los mecanismos a través de los cuales el cáncer pediátrico afecta la situación laboral de los padres, a fin de desarrollar modelos basados en la teoría que puedan orientar futuras investigaciones e identificar posibles objetivos de intervención. En segundo lugar, en lo que respecta a la investigación sobre intervenciones y la práctica clínica, los padres suelen experimentar cargas físicas y psicológicas considerables después de que a su hijo menor de edad se le diagnostique cáncer. Las intervenciones actuales se centran principalmente en aliviar la carga del cuidado y el malestar psicológico^(60,61)^, pero prestan menos atención a los cambios dinámicos en las necesidades de los padres y a su dificultad para equilibrar el trabajo y el cuidado. De hecho, las necesidades de los padres cambian significativamente a lo largo de la trayectoria del tratamiento del niño. Por lo tanto, las futuras investigaciones sobre intervenciones deberían centrarse en los desafíos específicos que enfrentan los padres en cada etapa del tratamiento de su hijo y brindar apoyo específico y personalizado para mantener el empleo y equilibrar las responsabilidades de cuidado. Por ejemplo, en la práctica clínica, el personal de enfermería se concentra actualmente principalmente en el tratamiento y los resultados de salud de los pacientes pediátricos con cáncer, descuidando a menudo los impactos psicológicos, financieros y laborales a largo plazo en las familias^(62,63)^. En este contexto, el personal de enfermería puede evaluar los conflictos de los padres entre el trabajo y el cuidado, así como sus necesidades de apoyo; brindar orientación individualizada; y coordinar recursos multidisciplinarios, como el trabajo social y la asesoría psicológica, para ayudar a las familias a equilibrar las responsabilidades del tratamiento, el cuidado y el empleo.

Desde una perspectiva de políticas públicas, las medidas de apoyo existentes siguen siendo insuficientes en términos de cobertura, accesibilidad y duración para satisfacer plenamente las necesidades continuas de los padres de niños con cáncer a lo largo de los procesos a largo plazo de tratamiento, rehabilitación y seguimiento^(64)^. El acceso a los recursos de salud y la continuidad de los servicios son fundamentales no solo para los resultados del tratamiento de los niños, sino también para la capacidad de los padres de mantener su empleo. Por lo tanto, se debe fortalecer la colaboración interdisciplinaria e intersectorial mediante la integración de recursos médicos, de enfermería, psicológicos, de trabajo social, de apoyo laboral y de seguridad social. Dichos esfuerzos pueden mejorar la continuidad y la coordinación de los servicios, y ayudar a los padres a lograr un equilibrio más sostenible entre el cuidado de los hijos y el trabajo. Además, en diferentes contextos nacionales y culturales, a menudo se espera que las madres asuman mayores responsabilidades de cuidado, y el cuidado prolongado puede aumentar su riesgo de interrupción laboral y limitar su desarrollo profesional^(10,31,44)^. Aunque las regulaciones laborales y los sistemas de protección social varían de un país a otro, medidas como la licencia por enfermedad remunerada, la licencia para el cuidado de familiares, los arreglos laborales flexibles, la protección del empleo, el seguro médico, la asistencia financiera y el apoyo caritativo pueden ayudar a aliviar el conflicto entre el trabajo y la familia y fortalecer la resiliencia económica de las familias^(65,66)^. Por lo tanto, las políticas futuras deberían mejorar aún más los sistemas de protección laboral y de protección social, y desarrollar mecanismos de apoyo más continuos, accesibles y equitativos para reducir los riesgos a largo plazo de inestabilidad financiera, interrupción laboral de los padres y malestar psicosocial.

### Limitaciones

Este estudio presenta varias limitaciones. En primer lugar, al tratarse de una revisión de alcance, los estudios incluidos variaron significativamente en cuanto a su diseño, las definiciones de los resultados laborales y los períodos de seguimiento, lo que limitó la comparabilidad entre estudios e impidió una síntesis cuantitativa. En segundo lugar, debe reconocerse la posibilidad de un sesgo de publicación. Este estudio incluyó estudios originales publicados, revisados por pares y en inglés, lo que pudo haber llevado a la omisión de literatura gris y de estudios publicados en otros idiomas. Además, solo se realizaron búsquedas en cinco bases de datos, lo que pudo haber dado lugar a la omisión de algunos estudios relevantes. En tercer lugar, aunque se incluyeron 21 estudios de 10 países, la evidencia procedía predominantemente de entornos de ingresos altos, con una representación limitada de países de ingresos bajos y medios. Dado que las normas culturales, las leyes laborales y los sistemas de protección social varían de un país a otro, es posible que los patrones de adaptación laboral de los padres no sean directamente transferibles entre diferentes contextos. Estas limitaciones pueden restringir la generalización de los hallazgos y la aplicabilidad más amplia de las implicaciones de política.

## CONCLUSIÓN

Este estudio muestra que tener un hijo menor de edad con cáncer puede causar alteraciones sustanciales y duraderas en el trabajo de los padres. Guiados por el Modelo Ecológico de la Salud, examinamos los factores que contribuyen a estas alteraciones en múltiples niveles, incluyendo rasgos personales, características psicológicas y de comportamiento, relaciones interpersonales, condiciones de vida y de trabajo, y el entorno normativo. Bajo la influencia de estos factores, los padres a menudo realizan ajustes significativos en su trabajo, tales como tomar licencias por enfermedad prolongadas, ajustar los horarios de trabajo, renunciar o cambiar de empleo. Las futuras investigaciones sobre intervenciones deberían centrarse en los desafíos que enfrentan los padres en las diferentes etapas del tratamiento de su hijo, ofreciendo intervenciones específicas y personalizadas relacionadas con el trabajo. Además, los responsables de la formulación de políticas deberían fortalecer el apoyo en materia de salud y trabajo para los padres de niños con cáncer, ayudándoles a aliviar sus cargas y permitiéndoles equilibrar mejor la vida familiar y laboral.

## Data Availability

El conjunto completo de datos que respalda los resultados de este estudio se publicó en el propio artículo.
